# Environmental adaptation in an asexual invasive insect

**DOI:** 10.1002/ece3.2894

**Published:** 2017-06-04

**Authors:** Jeffrey A. Lombardo, Joseph S. Elkinton

**Affiliations:** ^1^ Department of Environmental Conservation University of Massachusetts Amherst MA USA

**Keywords:** adaptation, cold hardiness, hemlock woolly adelgid, invasive species, parthenogenesis, supercooling

## Abstract

Parthenogenetic reproduction is generally associated with low genetic variance and therefore reduced ability for environmental adaptation, and this could limit the potential invasiveness of introduced species that reproduce asexually. However, the hemlock woolly adelgid is an asexual invasive insect that has spread across a large geographic temperature gradient in its introduced range. Consequently, this insect has shown significant variation in cold hardiness among populations. We hypothesized that the increased cold hardiness of northern populations represents an adaptation to the colder temperatures. To test this, we collected individual adelgid from populations spanning their invaded range and inoculated them into a common thermal environment. We then experimentally sampled the supercooling point of the progeny of these adelgids and compared these results with tests of the supercooling point of adelgid sampled directly from their source populations. The results showed that the same significant differences in supercooling that was found among geographically distinct populations existed even when the adelgid was reared in a common environment, indicating a genetic basis for the variation in cold hardiness. These findings support the hypothesis that the adelgid has adapted to the colder environment as it has expanded its distribution in its invaded range.

## 1 Introduction

Invasive species are a major ecological concern due to their detrimental effects on biodiversity, ecosystem functioning, disturbance regimes, etc. (Liebhold, MacDonald, Bergdahl, & Mastro, 1995; Pimentel et al., 2001). However, few species introductions result in established populations due to the numerous factors that inhibit the successful colonization of new habitat (Liebhold et al., 1995; Sakai et al., 2001). A major hurdle is that most introduced species are not well adapted to their new environment. This can result in reduced fitness and eventual extinction of the introduced population. For this reason, the genetic makeup of the founding population—including the amount of additive genetic variation—and consequently their ability to rapidly adapt to the new environment are important factors in both the successful colonization of new habitat and expansion of their distribution following establishment (Carroll, Dingle, Famula, & Fox, 2001; Lambrinos, 2004; Lee, 2002).

The process of environmental adaptation is driven by directional selection (Cano, Escarre, Fleck, Blanco‐Moreno, & Sans, 2008; Lambrinos, 2004; Maynard‐Smith, 1980), and a species response to selection is a function of their additive genetic variation (Fisher, 1930). Not all species respond to selection in the same manner. For example in asexually reproducing organisms, environmental adaptation presents a challenge because unlike sexual reproduction the lack of recombination reduces genetic variation under directional selection (Burger, 1999; Charlesworth, 1993; Crow, 1992). For this reason, it is reasonable to suggest that parthenogenetically reproducing organisms might be a lower risk for becoming invasive, particularly in regions where environmental variation is high. While previous studies have demonstrated adaptation in asexual organisms, the majority of these involved relatively stable conditions (Grapputo, Kumpulainen, Mappes, & Parri, 2005; Liu & Trumble, 2007; Llewellyn et al., 2003; Sandstrom, 1996; Sunnucks, Chisholm, Turak, & Hales, 1998). Few studies have experimentally tested the ability of an asexual invasive species to adapt to a stochastic environment. There is evidence of such adaptation in certain phenological traits of the asexual alpine grasshopper (*Chorthippus cazurroi*) when reared under variable conditions in the lab; however, these results were generally not evident in wild populations (Laiolo & Obeso, 2015). A more probable example is that of the invasive parthenogenetic insect, the hemlock woolly adelgid (*Adelgis tsugae* Annand). This insect has shown significant differences in cold hardiness among populations spanning a large geographic gradient of cold temperature extremes (Skinner, Parker, Gouli, & Ashikaga, 2003). However, because cold tolerance in insects is generally induced by exposure to progressively colder temperatures (Elkinton et al., 2016; Zachariassen, 1985), it is unclear whether the variation represents evolutionary adaptation (genetically based) or environmental acclimation (phenotypic). Subsequent work on the hemlock woolly adelgid by Butin, Porter, and Elkinton (2005) provided evidence of a genetic basis for the regional variation in cold tolerance in this insect. Their study was limited in spatial scale though and examined adelgid from just two locations (one from a northern population, and one from the Mid‐Atlantic region). Yet, combined these prior studies on this insect provide evidence of environmental adaptation despite its obligately asexual life history.

The purpose of this study was to experimentally test for adaptation in an asexual species, using the hemlock woolly adelgid as a model system. We focused on a specific functional trait, cold tolerance, which is an important adaptation to northern environments. Differentiating between adaptation and acclimation requires experimentally testing adelgid from across their geographic range with methods to control for the sources of variation (adaptation vs. acclimation). Therefore, we employed a robust method involving the artificial inoculation of adelgid from populations spanning their introduced range into a common thermal environment.

We used a quantifiable measure of cold hardiness, freeze avoidance, as our physiological trait. Freeze avoidance constitutes the prevention of ice crystal formation in subfreezing temperatures via the induction of cryoprotectants such as glycerol and other polyols within the insect hemolymph (Zachariassen, 1985). Production of cryoprotectants is a physiological response. While some degree of cold tolerance in insects is present constituently, a large portion is induced by exposure to increasingly cold temperatures. Generally, the quantity of cryoprotectants determines the level of protection (Lee, Chen, & Denlinger, 1987).

**Figure 1:**
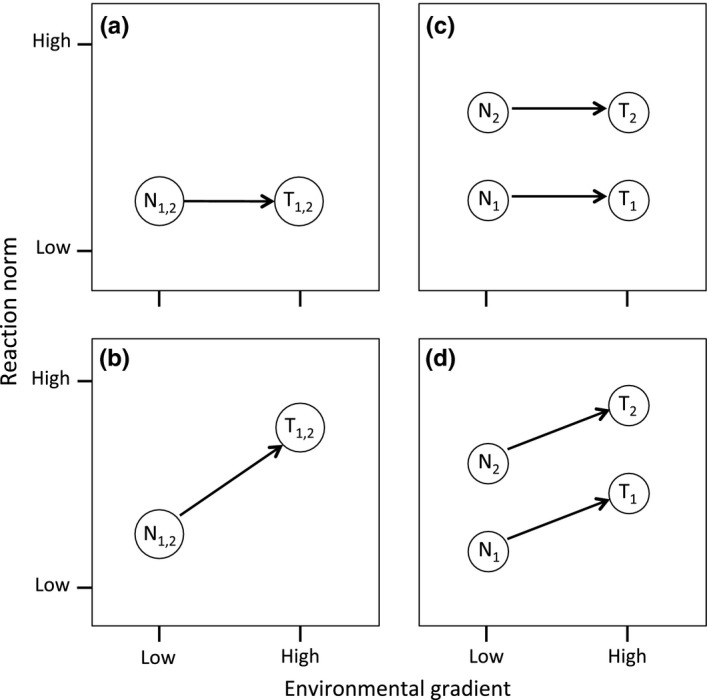
Four hypotheses for determining the influence of adaptation and environmental acclimation on a functional reaction norm, modified after Conover and Schultz (1995). *N*_i_ = individuals from a natural population, *T*_i_ = individuals from a transplant or common garden experiment. (a) No effect of environmental adaptation or acclimation. (b) No evidence of adaptation. Acclimatization in the direction of the environmental gradient. (c) Adaptation separates the two populations along the *y*‐axis. This is maintained in the common environment group. No effect of acclimation (connecting lines have slopes ≈ 0). (d) Both adaptation and environmental acclimation. Indicates a G × E interaction

We initiated a series of a priori hypotheses following from a modified version of those outlined by Conover and Schultz (1995, box 2) for a common garden style experiment. Our hypotheses are demonstrated in Figure 1 (boxes a–d, respectively) and cover four potential outcomes: (1) a null model where neither acclimation nor adaptation influences the physiological reaction norm, (2) variation in the reaction norm due to acclimation, with no evidence of adaptation, (3) adaptation as the sole source of variation in the reaction norm, and (4) both acclimation and adaptation influencing variation.

## 2 Methods

### 2.1 Model species

**Figure 2:**
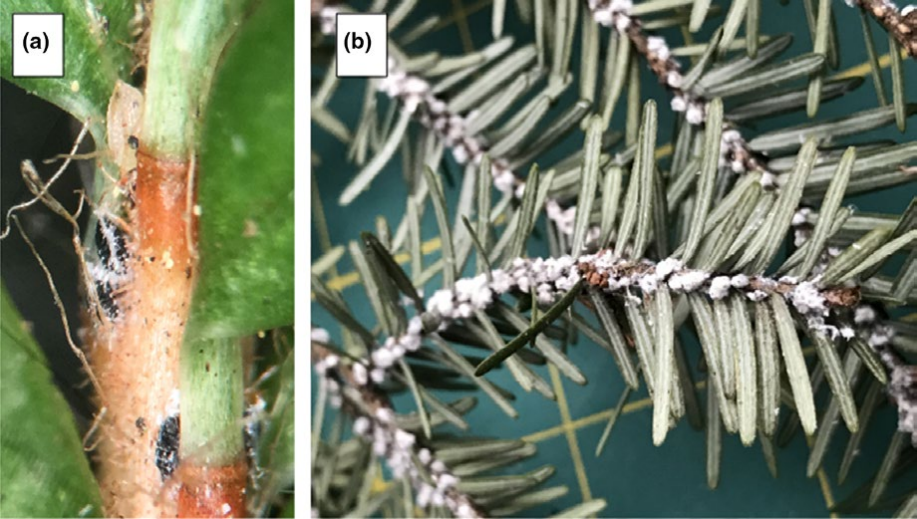
Early instar larvae of hemlock woolly adelgid attached to the base of hemlock leaves (a), and hemlock twigs covered with mature ovisacs (b). Photo A by R. Crandall used with permission

The hemlock woolly adelgid (Figure 2) is an invasive insect introduced to the eastern United States near Richmond VA, in 1951 from Japan (Havill, Montgomery, Yu, Shiyake, & Caccone, 2006). By the 1980s, it had spread to the New England states, and its range currently spans from southern Maine to northern Georgia. The insect is native to Asia as well as the Pacific Northwest of the United States (Havill et al., 2006). In both of these native habitats, the insect is largely benign. In its invaded range, however, the adelgid has caused widespread mortality of hemlock trees.

The adelgid produces two generations per year. The sistens generation hatch in early summer. They estivate most of the summer and resume development in the fall. Adult adelgid is nonmotile and are covered by protective waxy fibers resembling wool, hence the name (Figure 1b). They feed on tree synthate via a stylet inserted into the phloem tissue near the base of a needle. The adults of the sistens generation oviposit in late winter, and eggs of the progrediens generation hatch in early spring. By early summer, the progrediens have matured and produced eggs of the next sistens generation. Often, a percentage of the progrediens generation will mature into winged adult males called sexuparae. In Asia sexuparae produce a sexual generation. Sexual reproduction, however, is dependent upon a specific Asian species of spruce trees (*Picea spp*.) as an alternate host. In North America these species of spruce do not occur. Therefore, all North American populations are obligately asexual, and although sexuparae may still occasionally be produced, they do not contribute to future generations (McClure, 1989).

### 2.2 Regional variation in supercooling

To determine the regional variation in cold hardiness of hemlock woolly adelgid, we tested the supercooling point of individuals collected from 14 different sites spanning their introduced range. A list of the locations can be found in Table 1. Sites were sampled in February and March of 2015 and again in December of 2015. The samples consisted of small branches (25–35 cm in length) with a moderate–high density of live, apparently healthy adelgid ovisacs on both new and previous years branch growth (5–10/cm). To measure the supercooling point, an individual adelgid was selected from a sample branch. We removed the waxy outer covering (“wool”) and attached the individual to the end of a K‐type thermocouple sensor using clear packaging tape. The thermocouples were placed into a container submerged in a supercooling bath (Neslab RTE‐140), and the temperature of the bath was slowly reduced from a starting point of 5°C down to −35°C over the course of 3 hours (≈1°C change every 5 minutes). The temperature of each adelgid was recorded in one‐second intervals using a multi‐channel thermocouple recorder (Physitemp Inc., NJ, USA). When an adelgid freezes, the heat of fusion produces an obvious spike in the temperature. We used the temperature in the second prior to the thermal spike as the supercooling point. For each of the 14 sampling sites, we tested approximately 25 adelgids. All samples were tested within 48 hours of collection and were maintained at a constant 12°C for much of that time.

**Table 1:** Locations of adelgid sampling sites, their regional designation, sampling dates, experimental use, and the mean minimum yearly temperature for each site

Site	Region	Sample dates	Reg. source (RS) Com. garden (CG)	Mean min. temperature^a^
Mine Kill, NY	N	Dec, Feb, Mar	RS	−25.5
Shelburne Falls, MA	N	Dec, Feb	RS, CG	−22.3
Amherst, MA	N	Dec, Feb, Mar	RS, CG	−22.7
Quabbin Res., MA	N	Dec, Feb, Mar	RS, CG	−22.0
Taughannock Falls, NY	N	Dec, Feb, Mar	RS	−24.0
Del. Water Gap, NJ	A	Dec	RS, CG	−21.9
Kingston, RI	A	Dec, Feb	RS, CG	−19.1
Hamdon, CT	A	Dec, Mar	RS, CG	−19.0
Buzzards Bay, MA	A	Dec, Feb	RS	−18.7
Valley Forge, PA	A	Dec	CG	−18.9
Blacksburg, VA	S	Dec, Feb	RS, CG	−16.2
Powhatan, VA	S	Dec	RS	−14.4
Bent Creek, NC	S	Dec, Feb	RS, CG	−13.8
Kentland, VA	S	Dec, Feb, Mar	RS, CG	−15.7
Helen, GA	S	Dec, Feb, Mar	RS, CG	−12.5

a Average of yearly absolute minimum temperature for the period 2007–2016.

To analyze the data, each of the regional source populations was assigned to one of three geographic categories, specifically North, Atlantic, or South. These categories were based on the average minimum winter temperature of each site as determined using data from the National Climate Data Center. Sites with a mean minimum winter temperature <−22°C were classified as North, ≥−18 and ≤−21.9°C were classified as Atlantic, and >−18°C were labeled as South. A list of sites in each category is found in Table 1. The distributions (mean and standard deviation) of supercooling points for each region were compared for the February, March, and December sampling periods. In addition, a one‐way analysis of variance was used to test for differences in the means of each region. This was followed with a post hoc multiple comparisons test using a Tukey–Kramer HSD to determine pairwise differences. All analyses were completed using the R statistical software version 3.2.1 (R Core Development Team, 2015).

### 2.3 Common garden experiment

To separate the influence of environmental acclimation from the role of genetic adaptation in the cold hardiness of hemlock woolly adelgid, we transplanted gravid, sistens generation adelgids from 10 sites (latitudinal range: 34.7858–42.5719) across their introduced range into a common garden experiment (Table 1). It is important to note that in this experiment, the term common garden is referring to a common thermal environment. Within‐stand microsite variation of other environmental factors such as soil condition, light quality, slope, aspect, etc. were not standardized.

The experiment was set up in a naturally occurring eastern hemlock stand at the Quabbin Reservoir in western Massachusetts (42.4582, −72.3852). The stand is mostly free of adelgid infestation. The inoculations were completed in the first half of April 2015 on 66 hemlock trees in the stand. Each of the ten sites was inoculated onto multiple trees (~5–10 trees for each site), with only a single inoculation per tree. The inoculation procedure consisted of securing the samples to a healthy branch (~0.75 m of branch length) of a hemlock tree in the common garden experiment, covering the inoculated branches with a fine mesh bag to prevent the adelgid from spreading to nontarget branches, and letting the inoculation set for approximately 3 weeks. We used a quick visual inspection to ensure that the number of ovisacs inoculated were not vastly different. The eggs of these sistens adults gave rise to 1st instar crawlers of the progrediens generation, which established a new population of adelgid on our experimental trees in the common garden. Afterward the bags and the inoculum were removed.

To determine the cold hardiness of these adelgids, we collected samples from the common garden experiment in December of 2015. These samples represented the F_2_ sistens generation that arose from the progrediens that were established in the common garden the previous spring. A benefit of this method is that it can help to avoid potential complications due to maternal effects on cold tolerance, because the maternal generation never experienced extreme cold. Once samples were collected, we tested their supercooling point using the exact same procedures as outlined for the regional samples. As with the regional samples, those from the common garden were tested within 48 hr of collection and were maintained at a constant 12°C for most of that time. We had scheduled a second sampling date for February 2016; however, we were unable to complete the February sample due to an extreme cold temperature event (approx. −25°C, 13 February 2016) which resulted in >90% adelgid mortality in all samples at the common garden site.

To examine differences in the cold hardiness of the adelgid, we grouped the data by region of the source population (North, Atlantic, South) and examined the form and spread of the distributions (see Table 1). We also used a one‐way analysis of variance with *region* as a factor to determine differences in the means among regions. This was followed by a post hoc Tukey–Kramer HSD test to examine pairwise differences. Again, all analyses were completed using the R statistical software version 3.2.1 (R Core Team, 2015).

## 3 Results

### 3.1 Regional variation

**Figure 3:**
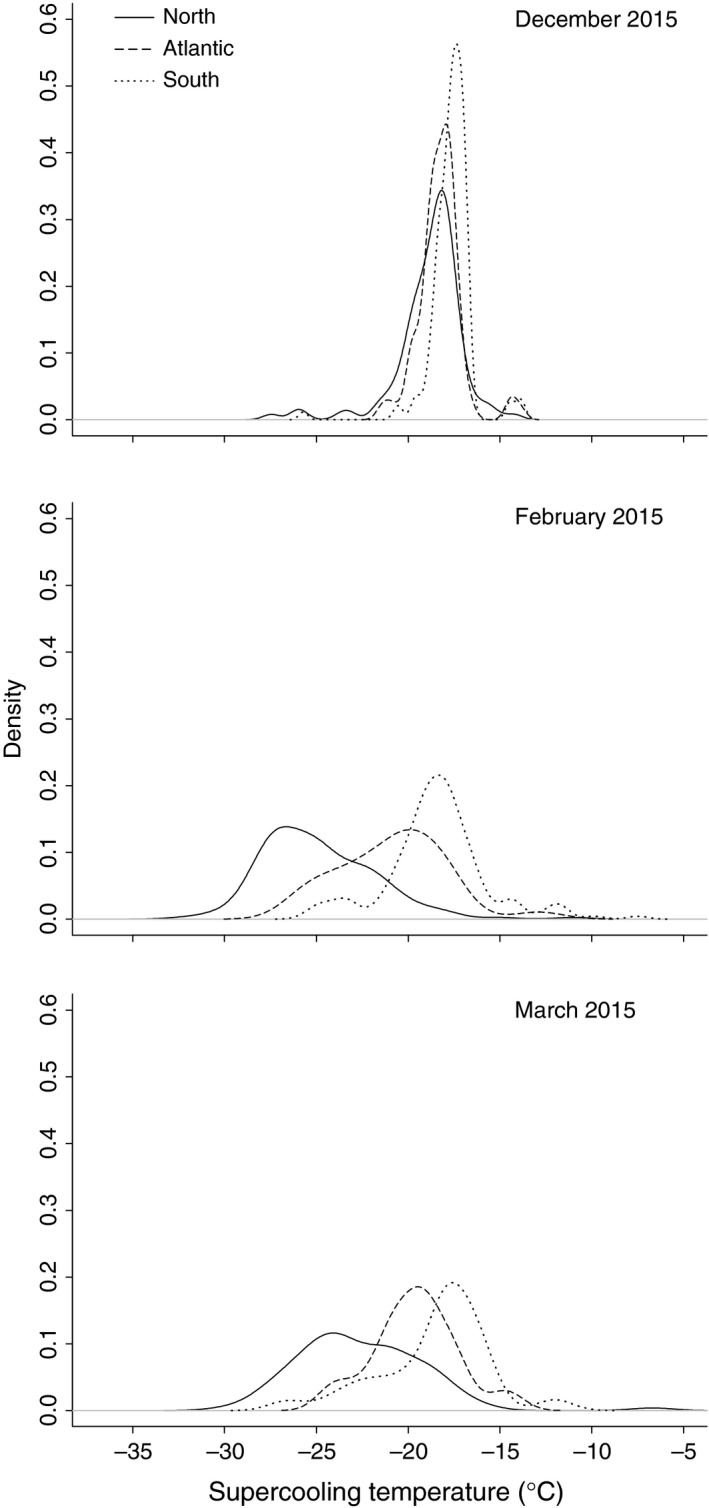
The distribution of supercooling points of hemlock woolly adelgid from three regions of their invaded range (North, Atlantic, South), sampled at three time periods

Lab tests of the supercooling point of hemlock woolly adelgid from three winter sampling events showed significant geographic variation, and each of the sampling dates showed the same north–south gradient of increasing cold tolerance (Figure 3). One‐way ANOVA results from the December 2015 data showed a significant difference in supercooling (*F*_2,332_ = 21, *p *<* *.001), and a post hoc test showed that all three regions were different from each other (not shown). The February data showed significant differences in mean supercooling (*F*_2,345_ = 191.3, *p *<* *.001), and again post hoc test showed all three were different from each other (not shown). The March data also showed significant differences among the three regions (*F*_2,219_ = 40.99, *p *<* *.001). Post hoc analysis, however, showed that the Atlantic and the South were not different from each other, although both were different from the North (not shown).

### 3.2 Common garden

**Figure 4:**
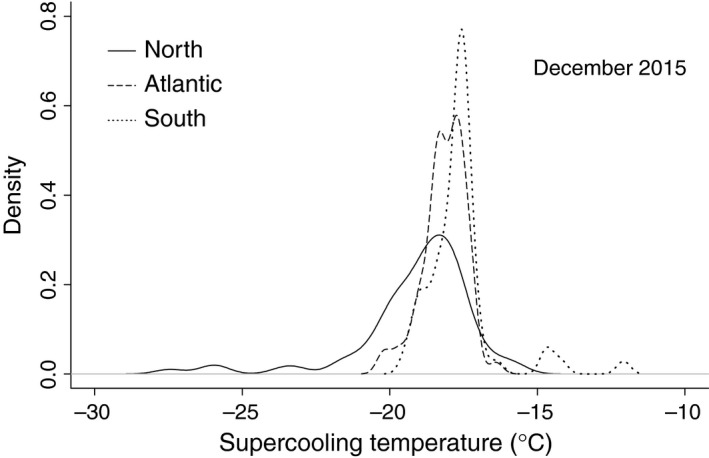
The distribution of supercooling points of hemlock woolly adelgid collected from source populations in three different regions of their introduced range and reared in a common environment showed significant differences between those from the Southern versus Northern populations, as well as the North versus Atlantic. Adelgids were inoculated into a common garden in spring of 2015 and sampled for supercooling point the following December

Data from the common garden was compiled into the same North, Atlantic, and South classifications as was used in the regional analyses, based on location of their source populations, with means (±*SD*'s) of −19.1 (±2.02), −18.1 (±0.72), and −17.6 (±1.20) for the North, Atlantic, and South, respectively (Figure 4). Despite these adelgids having been reared in a common environment, there was a significant effect of *Region* (*F*_2, 207_ = 18.66, *p *<* *.001), and post hoc analysis showed that all three of the regions were significantly different from each other.

### 3.3 Comparison of the common garden with the source populations

**Figure 5:**
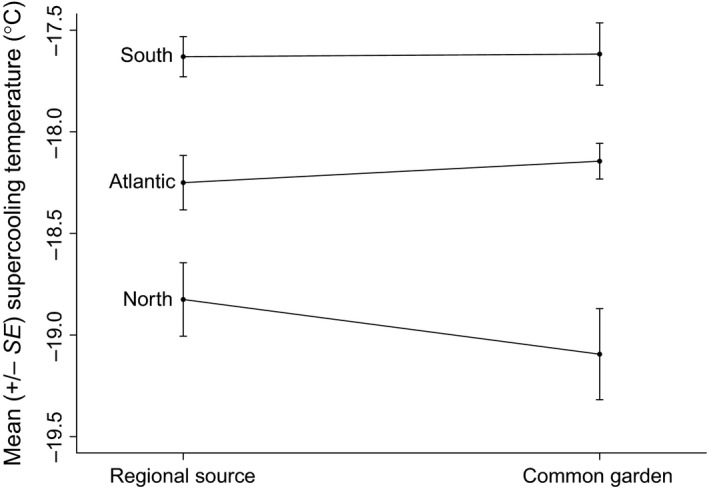
Significant differences in the supercooling point of adelgid from different source populations are maintained when they're reared in a common environment, indicating the populations have adapted to their regional environments. No significant differences in the pairwise comparisons among the three regions (connecting lines have slopes ≈ 0) indicate that environment does not explain these differences. See Figure 1 for an overview

The significant variation that was seen in the December 2015 sample of adelgid from throughout their introduced range when exposed to ambient conditions was also maintained when individuals from those same populations were reared in a common environment. This is evident by the approximately horizontal lines connecting the mean supercooling temperatures of adelgid from their regional source populations with those from the same populations from the common garden (Figure 5). We used a multi‐way ANOVA to test for differences in the main effects of *treatment* (source population, common garden), and *region* (North, Atlantic, South), as well as the *treatment × region* interaction. We found significant differences in both main effects, and no significant difference in the interaction term, indicating that all sites responded to the treatment in the same manner (Table 2). A follow‐up post hoc analysis showed no significant differences in the pairwise comparisons among *treatment* (Table 3). In other words, supercooling point of adelgid sampled from populations in the south was not different from southern adelgid reared in the common garden, and likewise for the other two regions. These results provide strong support for a genetic basis for the variation in cold tolerance among populations of this insect.

**Table 2:** Two‐way analysis of variance test for the effect of treatment (source populations vs. common garden), region (North, Atlantic, South) and the interaction of treatment and region on supercooling point of hemlock woolly adelgid in December 2015

Source	DF	MS	*F*	*p*
Treatment	1	18.26	8.54	<.001^a^
Region	2	77.15	36.09	<.001
Treatment × region	2	2.17	1.02	.3627
Error	539	2.14		

a Although the means of the two groups were significantly different, there were no within‐group differences (i.e., North from the source populations were not different from the North reared in the common garden, etc.) (Tukey HSD, α = 0.05).

**Table 3:** Tukey–Kramer HSD post hoc analysis of pairwise differences in the supercooling point of adelgid sampled from their source population versus those from the same populations when reared in a common environment

Comparison of source pop. versus common garden	Difference	Lower	Upper	*p* (adj.)
South	0.01	−0.63	0.65	.9999
Atlantic	0.11	−0.58	0.79	.9979
North	0.67	−0.49	1.83	.5697

## 4 Discussion

Data from the common garden experiment supported the hypothesis of a genetic basis for the variation in cold hardiness in the invasive hemlock woolly adelgid. This suggests that northern populations have adapted to the harsher winter environment in that region, as is evident by the lower supercooling point of northern adelgid. Despite covering a much more expansive region of the adelgids introduced range, our conclusions are essentially similar to that of Butin et al. (2005) who also used a common garden style experiment and found significant interactive effect of cold shock treatments and geographic region. While such adaptive processes had been previously documented in a number of different insect orders, there are few examples pertaining to strictly parthenogenetic insects. The greenhouse cockroach (*Pycnoscelus surinamensis*) is one such example. Parthenogentic populations of this insect have adapted to a broad range of environments and have become established on nearly every continent (Lundmark & Saura, 2006; Niklasson & Parker, 1994). However, its close association with humans is likely a large contributing factor in its wide geographic distribution (Niklasson & Parker, 1994). A couple well‐known examples of adaptive evolution in parthenogenetic species include the root‐knot nematode (*Meloidogyne sp.)*, (Castagnone‐Sereno, 2006), and the simultaneous evolution of multiple adaptive traits in clonal lab populations of *Daphnia Magna* (Boersma, Meester, & Spaak, 1999).

While winter temperature is the selective factor, it is not clear whether extreme cold tolerance was present in the founding population, or whether it was acquired via mutation in the invasive population. Butin et al. (2005) laid out a mathematical generalization for mutation to act as a source of adaptation. However, the acquisition of adaptive mutations in a strictly parthenogentic population can be problematic (Garrish & Lenski, 1998; Hill & Robertson, 1966; Wagner & Gabriel, 1990), and Butin et al. (2005) acknowledged this issue. A more parsimonious explanation is that genes conferring extreme cold tolerance were present in the founding population, and as the adelgid expanded its range northward the colder environment selected for the most cold‐tolerant of those individuals. In its native range, this insect exists across a gradient of thermal extremes similar to that of the eastern US. Therefore, it is reasonable to expect that such extreme cold tolerance could have been present in the founding population.

One of the concerns when testing for evidence of environmental adaptation is the potential for maternal effects to influence the results. Our experiment was designed to avoid maternal effects by using the progrediens generation to inoculate the common garden, and sampling from the subsequent sistens. This ensured that the maternal generation was not exposed to extreme cold temperatures, because eggs of the progrediens are laid in the spring and hatch in the early summer. The progrediens give rise to the sistens generation in early fall, and it was those sistens adults that we sampled throughout the winter. Of course this design does not guarantee that maternal effects were not present. Indeed, in some instances maternal effects have been shown to occur across generations (i.e., the F2 generation). This is sometimes referred to as grand maternal effects. For example, exposure to cold temperature in some *Drosophila* spp. can occasionally influence the cold hardiness of both the F1 and F2 generations, but the effect on the F2 generation is dependent upon specific environmental context (Watson & Hoffmann, 1995). Generally, grand maternal effects seem to be uncommon in most insects (Mousseau & Dingle, 1991; Mousseau and Fox 1998). We were unable to find any research that specifically tested for any type of maternal effects in the hemlock woolly adelgid; however, studies have shown an absence of grand maternal effects in the closely related Aphidoidae (Andrade & Roitberg, 1995; McLean, Ferrari, & Godfray, 2009; Via, 1991; Zehnder & Hunter, 2007). While we cannot rule out the possibility of grand maternal effects as an alternate explanation for our results, we are confident that such effects were unlikely to have occurred.

Environmental adaptation is a fundamental component of the invasion process for introduced species. What is not clear, however, is if adaptation will continue to allow the adelgid to expand its range further to the north, where host trees persist for hundreds of miles. There are a few scenarios by which the adelgid could continue its northern range expansion. First, it is possible that the adelgid is yet to reach their physiological limit of extreme cold tolerance. According to our results and those of others (Butin et al., 2005; Parker, Skinner, Gouli, Ashikaga, & Teillon, 1998; Skinner et al., 2003), the maximum cold tolerance for northern adelgid is between −30 and −35°C. Such temperatures are not uncommon minimum winter temperatures in the northern range of hemlock trees. Even at such extreme temperatures, however, some adelgid may survive (approx. 1% survival according to Skinner et al., 2003). As new populations of hemlock woolly adelgid can arise from a single individual (Tobin, Turcotte, & Snider, 2013), it is plausible that the adelgid could continue to expand its range. This would be a slow northward progression, compared to the rapid range expansion that occurred in warmer regions. Alternatively, a more cold‐tolerant ecotype could be introduced from their native range in Asia. Extreme cold temperatures in their native range are similar to those in the northern extent of hemlock trees in eastern North America. Finally, climate change‐related increases in minimum winter temperature could facilitate northward range expansion (Paradis, Elkinton, Hayhoe, & Buonaccorsi, 2008).

## Conflicts of interest

The authors declare no conflicts of interest.
